# The Classification of* Sini* Decoction Pattern in Traditional Chinese Medicine by Gene Expression Profiling

**DOI:** 10.1155/2016/8239817

**Published:** 2016-04-20

**Authors:** Hung-Tsu Cheng, Chaang-Ray Chen, Chia-Yang Li, Chao-Ying Huang, Wun-Yi Shu, Ian C. Hsu

**Affiliations:** ^1^Institute of Nano Engineering and Microsystems, National Tsing Hua University, No. 101, Section 2, Kuang-Fu Road, Hsinchu 300, Taiwan; ^2^Classics Chinese Medicine Clinic, Taipei 104, Taiwan; ^3^Department of Biomedical Engineering and Environmental Sciences, National Tsing Hua University, Hsinchu 300, Taiwan; ^4^Department of Genome Medicine, College of Medicine and Center for Infectious Disease and Cancer Research, Kaohsiung Medical University, Kaohsiung 807, Taiwan; ^5^Institute of Statistics, National Tsing Hua University, Hsinchu 300, Taiwan

## Abstract

We investigated the syndromes of the* Sini* decoction pattern (SDP), a common ZHENG in traditional Chinese medicine (TCM). The syndromes of SDP were correlated with various severe* Yang deficiency* related symptoms. To obtain a common profile for SDP, we distributed questionnaires to 300 senior clinical TCM practitioners. According to the survey, we concluded 2 sets of symptoms for SDP: (1) pulse feels deep or faint and (2) reversal cold of the extremities. Twenty-four individuals from Taipei City Hospital, Linsen Chinese Medicine Branch, Taiwan, were recruited. We extracted the total mRNA of peripheral blood mononuclear cells from the 24 individuals for microarray experiments. Twelve individuals (including 6 SDP patients and 6 non-SDP individuals) were used as the training set to identify biomarkers for distinguishing the SDP and non-SDP groups. The remaining 12 individuals were used as the test set. The test results indicated that the gene expression profiles of the identified biomarkers could effectively distinguish the 2 groups by adopting a hierarchical clustering algorithm. Our results suggest the feasibility of using the identified biomarkers in facilitating the diagnosis of TCM ZHENGs. Furthermore, the gene expression profiles of biomarker genes could provide a molecular explanation corresponding to the ZHENG of TCM.

## 1. Introduction

Traditional Chinese medicine (TCM) has been adopted to treat the Chinese for thousands of years on the basis of some classic Chinese medicine textbooks, including* Huang Ti Nei Jing* (“The Inner Cannon of Huangti”) [1],* Shénnóng Běn Cǎo Jīng* [2], and* Shang Han Za Bing Lun* (“Treatise on Cold Damage Disorders”) [3].* Shang Han Za Bing Lun* is a Chinese medical treatise written by Zhang Zhongjing before AD 220, at the end of the Han dynasty. The treatise is the oldest comprehensive clinical textbook in the world containing principles, methods, formulas, and medicine and is the clinical literature of TCM theory with practice. To obtain the appropriate decoction for a particular ZHENG requires a diagnostic system, namely, the Decoction ZHENG proposed in* Shang Han Za Bing Lun*. Through the methods of inspection, listening, smelling, inquiry, and palpation, physicians collect information on patients to identify their TCM ZHENGs according to their personal experience and then treat these patients with herbs, which is called decoction. The methods applied in TCM have been criticized as insufficiently scientific. To overcome this weakness, we conducted systems biology research to differentiate TCM ZHENGs.

Systems biology-based diagnostic principles can be used to support the relationship between TCM and current biomedicine [4, 5]. From the perspective of gene expression profile, TCM ZHENGs differentiation is closely related to gene polymorphisms and differences in gene expression profile. Therefore, applying advanced sequencing techniques and cDNA microarray studies can help to clarify the biological basis of TCM ZHENGs [4]. For example, by using microarray, real-time polymerase chain reaction, and enzyme-linked immunosorbent assay technologies,* Shen Yang Xu ZHENG* has been determined to be involved with the gene level [6] and may be primarily attributed to the insufficient activity of the mitogen-activated protein kinase (MAPK) pathway [7]. The* cold* ZHENG was discovered to be possibly caused by a physiological imbalance and disorder of metabolite processes by using microarray technology [8]. Human mRNAs in peripheral blood mononuclear cells (PBMCs) were extracted for microarray experiments to analyze differently expressed gene expressions for TCM* cold* and* hot ZHENG* differentiation [9–11].

In this study, we investigated the syndromes of* Sini* decoction pattern (SDP).* Sini* decoction was first mentioned in* Shang Han Za Bing Lun Sini* decoction consisting of three herbs: fresh* Aconitum carmichaelii*, dry* Zingiber officinale*, and* Glycyrrhiza uralensis*, which was characterized as a remedy to have essential effect of recuperating the patients from collapse. It was used to treat the syndrome of displaying coldness on the extremities, pulse feels deep or faint, continuous diarrhea with undigested cereal, profuse perspiration, abdominal fullness and distention, vomiting, and lethargy [12]. Based on the clinical applications of* Sini* decoction, SDP was then developed by Sun [13]. The syndromes of SDP were correlated with numerous symptoms, such as a slow pulse, coldness on the extremities, continuous diarrhea with undigested cereal, and severe diarrhea, but no common profile exists. According to our survey of 300 senior clinical TCM practitioners, we obtained a common profile for SDP: (1) pulse feels deep or faint and (2) reversal cold of the extremities. We collected SDP patients with these 2 symptoms. In this study, 12 patients with SDP and 12 non-SDP individuals were recruited from Taipei City Hospital, Linsen Chinese Medicine Branch, Taiwan.

We extracted the total mRNA of PBMCs from the 24 individuals for microarray experiments. Twelve individuals (including 6 SDP patients and 6 non-SDP individuals) were used as the training set to identify the biomarkers that were screened using the hypothesis test for distinguishing the SDP and non-SDP groups. The remaining 12 individuals (including 6 SDP patients and 6 non-SDP individuals) were used as the test set to examine whether the identified biomarkers can distinguish the SDP and non-SDP groups. The test results indicated that the gene expression profiling by the biomarkers could effectively distinguish the 2 groups by using a hierarchical clustering algorithm.

Our results suggested the feasibility of using biomarkers extracted from the gene expressions of PBMCs in facilitating the diagnosis of TCM ZHENGs. Furthermore, the gene expression profiles of biomarkers could provide the possible molecular mechanisms of related TCM ZHENGs.

## 2. Materials and Methods

### 2.1. Chinese Medicine Terminology

All Chinese medicine terms used in this paper were in accordance with the* International Acupuncture Nomenclature* (IAN) proposed by WHO in 1991; if not available in IAN, the standard nomenclature proposed in the* WHO International Standard Terminologies on Traditional Medicine in the Western Pacific Region* [14] was adapted. These translated Chinese medicine terms are presented in italics in this paper.

### 2.2. Human Participants

The syndromes of SDP were associated with a slow, deep, and faint pulse that disappeared, coldness on the extremities, severe diarrhea, continuous diarrhea with undigested cereal, generalized pain, abdominal fullness, cold-fluid retention on the diaphragm, profuse sweating, severe spasms in the feet, tight clamping of the extremities, and reversal cold in both the hands and feet; however, no common profile exists. To obtain a common profile for SDP, we distributed questionnaires to 300 senior clinical TCM practitioners. Our questionnaire listed 41 symptoms related to SDP. In the survey, each senior clinical TCM practitioner assigned a score to each symptom, indicating the correlation between the symptom and SDP from high to low (5, 4, 3, 2, 1, and 0). We identified symptoms that collectively constituted a common SDP profile according to the frequency with which participating TCM practitioners assigned a score of 5 to the symptoms; the frequency threshold was set at higher than 35% of the returned surveys. We recruited SDP patients with the aforementioned symptoms. In this study, 12 SDP patients and 12 non-SDP individuals from Taipei City Hospital, Linsen Chinese Medicine Branch, Taiwan, were recruited. All of the individuals provided informed consent. The definition of healthy subject was diagnosed by western medicine rather different from TCM. It is difficult to diagnose healthy subject. Therefore, we consider substituting non-SDP patients for healthy subject. We try our best to collect non-SDP patients who are alike healthy subjects.

### 2.3. Blood Sample Collection

We phlebotomized 30 mL of human peripheral blood from each donor and then separated the blood into three 10 mL plastic whole blood tubes with spray-coated K2EDTA (Becton Dickenson, Franklin Lakes, NJ, USA). The blood was transferred to a 50 mL centrifuge tube. The PBMCs were then prepared from the 30 mL of peripheral blood through Ficoll-Hypaque density gradient centrifugation. The PBMCs were added to an RNA*later* RNA Stabilization Reagent (Qiagen, Valencia, CA, USA) and then placed into a bottle with liquid nitrogen. All of these procedures were performed immediately after the blood was collected at the laboratory of Taipei City Hospital, Linsen Chinese Medicine Branch, Taiwan. The isolated PBMCs were frozen, stored in a liquid nitrogen freezer, and transported to the microarray experimental laboratory at National Tsing Hua University, Hsinchu, Taiwan, for further microarray experiments.

### 2.4. RNA Extraction and Microarray Hybridization

We extracted total RNA by using the RNeasy Mini Kit according to the manufacturer's protocol (Qiagen, Valencia, CA, USA). Total RNA quantity and quality were assessed using an ultraviolet absorption spectrophotometer at 260 and 280 nm. Subsequently, *α*- and *β*-globin mRNA were reduced from a portion of the total RNA samples by using the GLOBINclear*™* human kit (Ambion, Austin, TX, USA), according to the manufacturer's instructions, at the recommended start quantity of 10 *μ*g of total RNA. The quality of total RNA was evaluated using the Agilent 2100 bioanalyzer with the RNA 6000 Nano LabChip kit (Agilent Technologies, Palo Alto, CA, USA). Before reverse transcription, each RNA sample was spiked with a mixture of Arabidopsis mRNAs, which we called doping control. Fluorescence-labeled (Cy3, Cy5-3DNA Capture Reagent) cDNA was conducted using the 3DNA Array 900 labeling kit according to the manufacturer's protocols (Genisphere, Hatfield, PA, USA). Reverse transcription was performed using SuperScript II (Invitrogen, Carlsbad, CA, USA). Hybridization was performed at 65°C in a water bath for 16 to 18 h, and arrays were washed according to the manufacturer's protocol (Corning Life Sciences, New York, NY, USA). The arrays were scanned using GenePix 4000B scanner (Axon Instruments, Foster City, CA, USA).

### 2.5. Microarray Fabrication

A total of 9600 human cDNA clones were purchased from Incyte Genomics (Wilmington, DE, USA). These cDNAs were randomly selected from the IMAGE library. Resequencing was performed on these clones, and 7334 cDNAs were verified to have corrected DNA sequences. These 7334 sequence-verified human cDNAs (Incyte Genomics) and 10 Arabidopsis cDNAs (SpotReport*™* cDNA Array Validation System; Stratagene, La Jolla, CA, USA), serving as spike-in controls, and one housekeeping gene (*β*-actin), serving as a positive control, were arrayed on Corning UltraGAPS slides (Corning, Corning, NY, USA). These 7334 human cDNAs and 96 Arabidopsis cDNAs and housekeeping genes were spotted in quadruplicate on each array making a total of 32248 spots to enhance the statistical confidence in the gene expression data. Each array had 32,448 spots. The arrays were postprocessed according to the Corning UltraGAPS Coated Slides instruction manual.

### 2.6. Experimental Design

**Figure 1:**
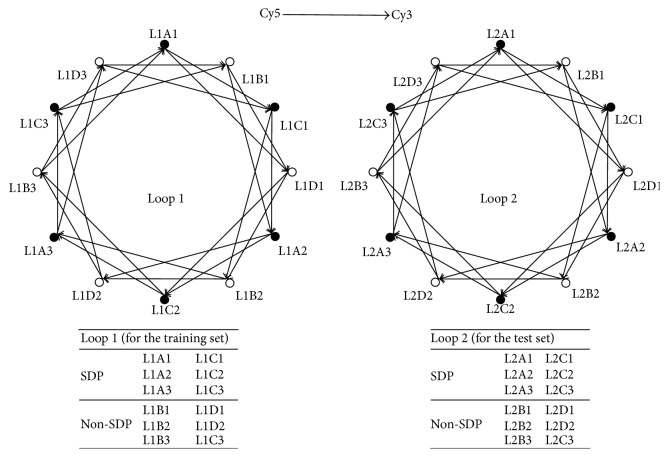
Experimental designs of Loop 1 (for the training set) and Loop 2 (for the test set). Each arrow indicates a microarray hybridization experiment. The arrow heads and tails represent samples labeled with Cy5 and Cy3, respectively. The black solid circles stand for the SDP samples and the open circles stand for non-SDP samples. Loop 1 is the experimental design for the training set of 12 individuals. Loop 2 is the experimental design for the test set of the other 12 individuals. The annotations of samples in Loop 1 and Loop 2 are shown below each loop.

Loop design, a type of statistical microarray experimental design, was adopted to construct an optimal design. The basic principles of optimal design are a balance among the factors, approximately equal sampling of varieties, and minimal distance between pairs of varieties [15]. Samples from 24 individuals were hybridized on 48 arrays based on the 2 loop designs presented in Figure 1. The individuals of samples L1A1, L1A2, L1A3, L1C1, L1C2, L1C3, L2A1, L2A2, L2A3, L2C1, L2C2, and L2C3 were SDP patients, and those of samples L1B1, L1B2, L1B3, L1D1, L1D2, L1D3, L2B1, L2B2, L2B3, L2D1, L2D2, and L2D3 were non-SDP individuals. Each arrow in the figure indicates a microarray hybridization experiment. The arrow heads and tails represent samples labeled with Cy5 (3DNA Capture Reagent) and Cy3 (3DNA Capture Reagent), respectively. The black solid circles stand for the SDP samples and the open circles stand for non-SDP samples. We used 12 individuals in Loop 1 experiments as the training set and the remaining 12 individuals in Loop 2 experiments as the test set.

### 2.7. Microarray Data Analysis and Statistical Analysis

Microarray data preprocessing, normalization, and statistical analysis were performed using the bioinformatics software suite Tsing Hua Engine for Microarray Experiment [16].

Spot-screening rules were applied to screen invalid spots on these arrays. The spot-screening rules for the data of Loop 1 (the training set) were as follows: (1) exclude the spots defined as “flag bad” or “absent” in all GenePix Results (GPR), (2) exclude the spots with diameters less than 100 *μ*m, (3) exclude the spots with a coefficient of variation (CV) of pixel intensity of over 100% in both channels, and (4) exclude the spots with signal to noise ratios (SNRs) in both channels were less than 2 in the training set microarray experiments. The SNR is defined as (*S* − *B*)/*B* (*S*: mean of pixel intensities of the signal; *B*: median pixel intensity of the background). The logarithm of the ratios for all valid spots on each array was normalized using global lowess normalization [17]. After the data were preprocessed, the normalized log ratios of the fluorescence intensity of each cDNA spot were analyzed using a log linear model, which was described in our previous study [18], to obtain the least-squares estimates $\hat{\lambda_{1}}$, $\hat{\lambda_{2}}$, $\hat{\lambda_{3}}$, $\hat{\lambda_{4}}$, $\hat{\lambda_{5}}$, $\hat{\lambda_{6}}$, $\hat{\lambda_{7}}$, $\hat{\lambda_{8}}$, $\hat{\lambda_{9}}$, $\hat{\lambda_{10}}$, $\hat{\lambda_{11}}$, and $\hat{\lambda_{12}}$ for the relative expressions among all of the samples. The relative expressions of sample *i* are defined as (expression of sample *i*)/(geometric average of the expression of all samples), for each gene, where *i* runs from 1 to 12. For the training set microarray data, 4951 genes satisfied the selection criteria of the spot-screening rules and log linear model. The selection criteria for the data of Loop 2 (the test set) are described in the subsequent subsection. The microarray data is available at GEO (GSE67090).

### 2.8. Searching Biomarkers to Distinguish SDP

We used Loop 1 experiments as the training set and Loop 2 experiments as the test set. For the 4951 genes of Loop 1 (the training set), 192 genes were identified using an *F* test with null hypothesis *H*_0_: ($\hat{\lambda_{1}}+\hat{\lambda_{3}}+\hat{\lambda_{5}}+\hat{\lambda_{7}}+\hat{\lambda_{9}}+\hat{\lambda_{11}})-(\hat{\lambda_{2}}+\hat{\lambda_{4}}+\hat{\lambda_{6}}+\hat{\lambda_{8}}+\hat{\lambda_{10}}+\hat{\lambda_{12}})=0$, at a Bonferroni-adjusted significance level of 0.05/4951, where $\hat{\lambda_{1}}$, $\hat{\lambda_{3}}$, $\hat{\lambda_{5}}$, $\hat{\lambda_{7}}$, $\hat{\lambda_{9}}$, and $\hat{\lambda_{11}}$ were the least-squares estimates for the relative expressions of the SDP samples and $\hat{\lambda_{2}}$, $\hat{\lambda_{4}}$, $\hat{\lambda_{6}}$, $\hat{\lambda_{8}}$, $\hat{\lambda_{10}}$, and $\hat{\lambda_{12}}$ were the least-squares estimates for the relative expressions of the non-SDP samples.

The 192 biomarker genes were then used to distinguish the SDP and non-SDP groups of the test set. We applied the selection criteria to filter bad spots to perform an effective normalization and a log linear model. In order to include additional available genes to be tested, we relaxed the selection criteria of the spot-screening rules for the data of Loop 2 (the test set) as follows: (1) exclude the spots defined as “flag bad” or “absent” in all GPR files, (2) exclude the spots with diameters less than 50 *μ*m, (3) exclude the spots with a CV of pixel standard deviation of over 150% in both channels, and (4) exclude the spots with SNRs in either channel were less than 1 in the test microarray experiments. The logarithm of the ratios for all valid spots on each array was normalized under the same normalization conditions as those for the training set. We then used hierarchical clustering to verify whether the gene expression profiles of the 192 biomarker genes could classify the 12 test samples into 2 groups and how well they could distinguish the SDP patients and non-SDP individuals.

### 2.9. Functional Annotation and Enrichment Analysis

Functional annotation and enrichment analysis were accessed through the database for annotation, visualization, and integrated discovery (DAVID) [19], which performed a modified Fisher's exact test to identify overrepresented functions. DAVID is a widely employed web tool that provides a rapid means to understand the biological meaning of a long list of genes of interest. For any gene list, DAVID can map and convert identifiers (IDs) and accessions among different gene IDs. A long gene list can be reduced into biological theme-related groups of genes, for example, gene ontology (GO) [20] and the Kyoto Encyclopedia of Genes and Genomes (KEGG) pathway [21].

The Entrez gene IDs of 192 biomarker genes were used as the gene ID for bioinformatics analysis. Biological processes of GO terms were used for functional annotation and enrichment analysis of these biomarker genes.

## 3. Results

### 3.1. Questionnaires to Obtain a Common Profile for SDP

**Figure 2:**
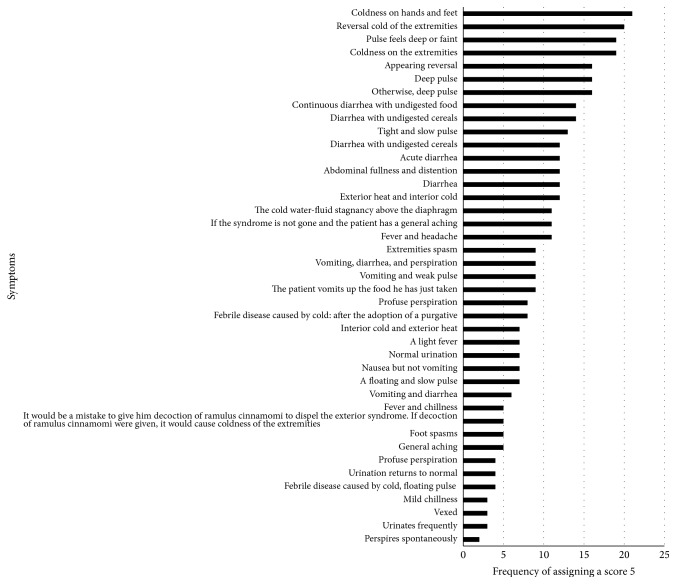
Frequency with which survey symptoms were assigned a score of 5 in determining the SDP profile.

We distributed questionnaires to 300 senior clinical TCM practitioners, and 43 questionnaires were returned. Our questionnaire listed 41 symptoms related to SDP. In the survey, each senior clinical TCM practitioner assigned a score to each symptom, indicating the correlation between the symptom and SDP, from high to low (5, 4, 3, 2, 1, and 0). Based on the frequency with which participating TCM practitioners assigned a score of 5 to the symptoms, we identified symptoms that collectively constituted a common SDP profile (Figure 2). The frequency threshold was set at higher than 35% of the 43 returned surveys (i.e., symptoms assigned a score of 5 by at least 16 surveys). This process revealed 7 symptoms to form our symptom profile of SDP patients. By grouping related symptoms, we obtained a common profile for SDP as 2 sets of symptoms: (1) pulse feels deep or faint and (2) reversal cold of the extremities. We recruited SDP patients with the 2 sets of symptoms. In this study, 12 SDP patients and 12 non-SDP individuals were recruited from Taipei City Hospital, Linsen Chinese Medicine Branch, Taiwan. All of the participants provided informed consent.

### 3.2. Identifying Biomarker Genes from the Training Set Microarray Experiment

**Figure 3:**
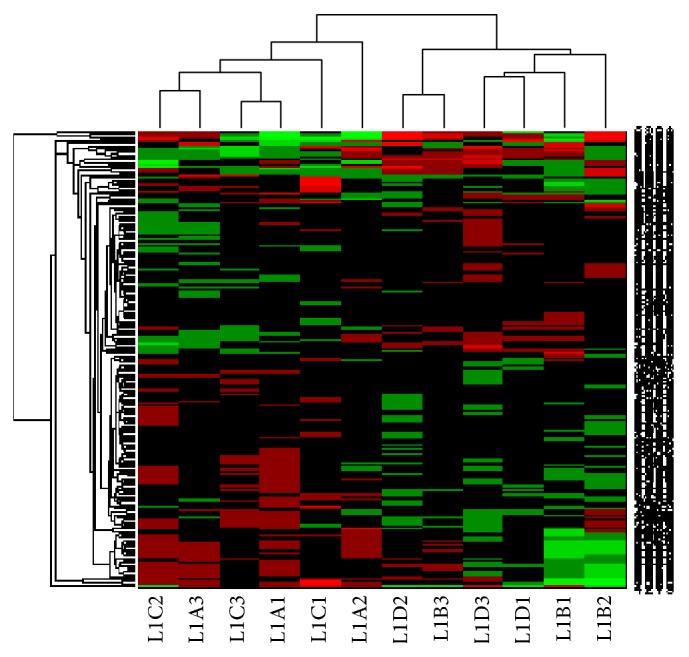
192 genes, identified using the *F* test at a Bonferroni-adjusted significance level of 0.05/4951, were clustered into 2 groups. By applying hierarchical clustering with Spearman distance and average linkage, the 12 training samples were appropriately separated into 2 groups according to the 192 marker genes. Samples L1C2, L1A3, L1C3, L1A1, L1C1, and L1A2 comprised the SDP group, and samples L1D2, L1B3, L1D3, L1D1, L1B1, and L1B2 comprised the non-SDP group. In this figure, the gene expression value used to do the clustering is the relative expressions of the labeled sample.

On the basis of the results for the 4951 genes of Loop 1 (the training set), 192 genes were identified (Table 1) using an *F* test with null hypothesis *H*_0_: $(\hat{\lambda_{1}}+\hat{\lambda_{3}}+\hat{\lambda_{5}}+\hat{\lambda_{7}}+\hat{\lambda_{9}}+\hat{\lambda_{11}})-(\hat{\lambda_{2}}+\hat{\lambda_{4}}+\hat{\lambda_{6}}+\hat{\lambda_{8}}+\hat{\lambda_{10}}+\hat{\lambda_{12}})=0$, at a Bonferroni-adjusted significance level of 0.05/4951. By applying hierarchical clustering with Spearman distance and average linkage, the 12 training samples were appropriately separated into 2 groups, as shown in Figure 3. In Figure 3, the gene expression value used to do the clustering is the relative expressions of the labeled sample.

### 3.3. Distinguishing Power of the Biomarker Genes to the Test Set Microarray Experiment

**Figure 4:**
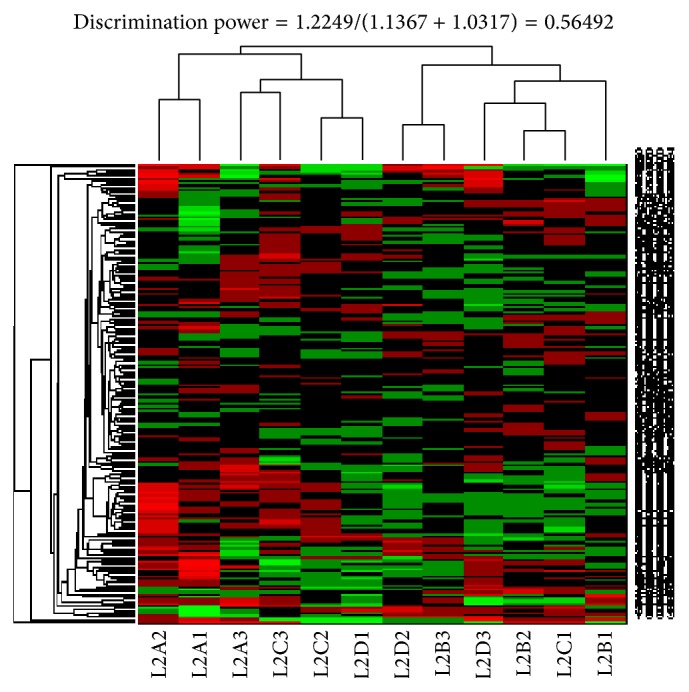
Hierarchical clustering results for the 12 test samples. Under the same clustering conditions (i.e., hierarchical clustering with Spearman distance and average linkage), the gene expression profiles of the 192 marker genes classified the 12 test samples into 2 groups. One group consisted of the L2A2, L2A1, L2A3, L2C3, L2C2, and L2D1 samples and the other consisted of the L2D2, L2B3, L2D3, L2B2, L2C1, and L2B1 samples. Except for the L2D1 and L2C1 samples, all of the samples were correctly grouped into SDP and non-SDP groups. In this figure, the gene expression values that used to do the clustering are the relative expressions of the labeled sample.

The 192 biomarker genes identified in the training set experiments were then used to test the ability to distinguish the SDP patients and non-SDP individuals in the test set. To include additional available genes to be tested, we relaxed the selection criteria of the spot-screening rules for the data of the test set (Loop 2). The logarithm of the ratios for all valid spots on each array was normalized under the same normalization conditions as those for the training set. We then used hierarchical clustering to verify whether the gene expression profiles of the 192 biomarker genes could classify the 12 test samples into 2 groups and how well they could distinguish the SDP patients and non-SDP individuals. Under the same clustering conditions (i.e., hierarchical clustering with Spearman distance and average linkage), Figure 4 showed that the 192 biomarker genes can classify the 12 test samples into 2 groups. One group comprised L2A2, L2A1, L2A3, L2C3, L2C2, and L2D1 samples and the other comprised L2D2, L2B3, L2D3, L2B2, L2C1, and L2B1 samples. Except for L2D1 and L2C1 samples, all of the samples were correctly grouped into SDP and non-SDP groups. In Figure 4, the gene expression value used to do the clustering is the relative expressions of the labeled sample. The microarray data of 4 biomarker genes from 7 samples were confirmed by quantitative PCR (qPCR). The correlation coefficients *r* between microarray data and qPCR for each gene are 0.73, 0.83, 0.93, and 0.76 (Appendix A).

### 3.4. Functional Annotation and Enrichment Analysis Results for the 192 Biomarker Genes

The functional annotation and enrichment analysis were accessed using DAVID. Seventy-eight GO terms were enriched with a *P* value less than 0.05. All enriched GO terms are listed in Appendix B. 78 GO terms were enriched with a *P* value of less than 0.05. We further applied 2 stringent criteria to filter the high-ranked GO terms: (1) GO terms containing more than 10 genes and (2) a *P* value of less than 0.01. Consequently, 11 GO terms were identified (Table 2).

Table 2 showed that the 11 GO terms are substantially correlated with immune, inflammation, and hemopoiesis functions. In detail, the immune function includes defense response, immune response, response to bacterium, and immune system development. The inflammation function includes cytokine production regulation, behavior, cell activation, and cellular homeostasis. The hematopoiesis function includes hemopoietic or lymphoid organ development and hemopoiesis.

## 4. Discussion

Of the 4951 genes of the training set, 192 genes were identified using an *F* test with null hypothesis. By applying hierarchical clustering with Spearman distance and average linkage, the 12 training samples were appropriately separated into SDP and non-SDP groups. The 192 biomarker genes were then used to distinguish the SDP patients and non-SDP individuals in the test set. Under the same clustering conditions, the 12 test samples were clustered into 2 groups. One group comprised L2A2, L2A1, L2A3, L2C3, L2C2, and L2D1 samples and the other comprised L2D2, L2B3, L2D3, L2B2, L2C1, and L2B1 samples. Except for L2D1 and L2C1 samples, all of the samples were correctly grouped into the SDP and non-SDP groups. Thus, we reviewed the original information of these 2 samples. L2C1 sample (classified as SDP according to TCM but clustered into the non-SDP group by using biomarkers) had a deep, rough, and rapid pulse and reversal cold of the extremities. Among the symptoms of L2C1 sample, reversal cold of the extremities matched the selected symptom of SDP; however, a deep, rough, and rapid pulse was inconsistent with a deep or faint pulse, a selected symptom for SDP. L2D1 sample (categorized as non-SDP according to TCM but clustered into the SDP group by using biomarkers) had a deep and tight pulse, which was particularly deep at the* guan* site (the guan is immediately central to the radial styloid at the wrist, where the tip of the physician's middle finger is placed), and reversal cold of the extremities. Among the symptoms of L2D1 sample, a deep and tight pulse, particularly at the guan site, did not meet the symptom of a deep or faint pulse, and the sample was thus classified as non-SDP according to TCM. However, reversal cold of the extremities matched the selected symptom of SDP. These findings indicate that, in classifying these 2 samples into SDP and non-SDP groups, uncertain areas exist. Nevertheless, determining biomarkers by using the mRNA profiling method can facilitate identifying the SDP group. Therefore, the traditional dialectical method of TCM is quite trustworthy.

The results of the functional annotation and enrichment analysis of 192 biomarker genes revealed that the 11 GO terms were substantially correlated with immune, inflammation, and hemopoiesis functions. Among the symptoms of SDP, a deep or faint pulse implied the insufficiency of *Qi* (in the field of medicine, *Qi* refers both to the refined nutritive substance that flows within the human body and to its functional activities). Insufficiency of *Qi* indicates the body's resistance against disease is weak. Pathogens can invade the human body easily, and viruses and bacteria could then cause inflammation easily.

## 5. Conclusion

Our results provided the biomarker genes of SDP, which was first mentioned in* Shang Han Za Bing Lun*. The results provide molecular evidence for TCM in the ZHENGs differentiation of SDP. Using mRNA profiling experiments on patients' PBMCs to discover biomarker genes to test an unknown group of individuals can effectively identify a specific ZHENG. We hope that the mRNA profiling method will become a model for TCM physicians in diagnosis and assist TCM practitioners in evaluating the curative effect of the medication.

## Figures and Tables

**Figure 5:**
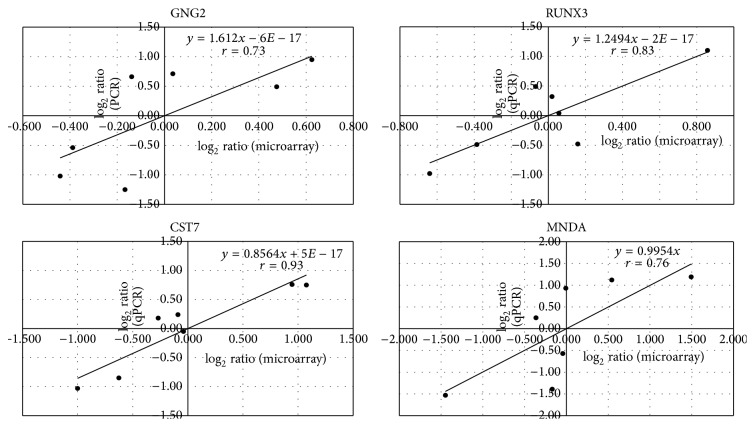
The correlation coefficients *r* for each gene are 0.73 (GNG2), 0.83 (RUNX3), 0.93 (CST7), and 0.76 (MNDA).

**Table 1:** The 192 biomarker genes identified using an *F* test with null hypothesis *H*_0_: $(\hat{\lambda_{1}}+\hat{\lambda_{3}}+\hat{\lambda_{5}}+\hat{\lambda_{7}}+\hat{\lambda_{9}}+\hat{\lambda_{11}})-(\hat{\lambda_{2}}+\hat{\lambda_{4}}+\hat{\lambda_{6}}+\hat{\lambda_{8}}+\hat{\lambda_{10}}+\hat{\lambda_{12}})=0$, at a Bonferroni-adjusted significance level of 0.05/4951.

Gene name				
ABHD3	ABI2	ACSM2B	ACTB	AGFG1
AKAP9	ARG1	ARID5A	ATP1B3	ATP6AP2
ATP6V0B	BLZF1	BPGM	C11orf79	C12orf24
C12orf32	C17orf91	C6orf106	CAPN1	CBARA1
CCL18	CCL5	CD1D	CD24	CD40
CD44	CD83	CD8A	CD93	CDC42
CDC42SE2	CLDN1	COMT	COX10	CP
CREM	CST7	CXCL9	DEFA1	DEFA3
DEFA4	DENND2D	DGCR6L	DLG2	DUSP6
EEF2	EIF3E	EIF3I	EPAS1	EXOSC9
FAM113B	FAM175B	FCER1A	FLNB	FMOD
FOLR3	FOS	FOSB	FYN	GCA
GIMAP1	GLRX	GNG11	GNG2	GNLY
GPR56	GSTA2	GTF2E1	GZMA	H3F3B
HERPUD1	HIST1H2AC	HIST1H2BD	HIST1H2BG	HIST1H2BJ
HIST2H2AA3	HLA-A	HLA-DMA	HLA-DQB1	HLA-DRB1
HLA-G	Hs.282050	Hs.467411	Hs.534061	Hs.586682
Hs.591807	Hs.599359	Hs.655903	Hs.674438	Hs.88605
HSP90B1	HSPA1A	HTATIP2	HTR7P1	ID2
IFIT1	IL10RA	IL15RA	IL1B	IL32
JARID1C	KIF3C	KLF10	KLF6	LCP1
LNP1	LOC400027	LOC729776	LTB	LYRM1
MAPK13	MBP	MCM3AP	MEST	MLH3
MNDA	MRPL3	MRPL49	MTMR6	MYL6
NCRNA00084	NHEDC2	NPY	NUCB1	NUDT16
NUP93	ORM1	PACS1	PCDH8	PDE4B
PEA15	PF4	PLAUR	PNLIPRP2	POLR1C
POLR2D	POU2F2	PPBP	PPP1R12B	PPP1R7
PPP2R2A	PPRC1	PPT1	PRL	PSMC6
RAB4A	RARRES3	RGS18	RGS2	RHOQ
RNF40	RNPEP	RPL41	RPS11	RPS24
RPS27	RPS4X	RPS4Y1	RUNX3	S1PR5
SEC22B	SELL	SERPINE2	SFRS1	SH2B3
SH3D19	SHCBP1	SLC2A3P1	SOD2	SPTY2D1
SRGN	STX8	SYTL2	TADA3L	TARP
THBS1	TIMP1	TMEM176A	TNFAIP8L2	TPCN1
TRA@	TRAPPC1	TRMT11	VIM	VPS13D
WDR42A	WIZ	XPNPEP1	YARS	Unknown^*∗*^
Unknown^*∗*^	ZNF286A			

^*∗*^The sequence of the clone was not yet identified in the BLAST.

**Table 2:** The 11 enriched GO terms for 192 biomarker genes (sorted according to *P* value).

GO term	Description	# gene	*P* value
GO:0006952	Defense response	24	0.000000
GO:0006955	Immune response	24	0.000002
GO:0009617	Response to bacterium	11	0.000088
GO:0001817	Regulation of cytokine production	11	0.000172
GO:0048534	Hemopoietic or lymphoid organ development	13	0.000562
GO:0030097	Hemopoiesis	12	0.000999
GO:0002520	Immune system development	13	0.001183
GO:0007610	Behavior	14	0.004162
GO:0006412	Translation	12	0.004210
GO:0001775	Cell activation	11	0.008090
GO:0019725	Cellular homeostasis	13	0.009574

**Table 3:** The correlation plots between microarray data and qPCR data.

Genes	Samples	Microarray data		qPCR data	
		log_2_ of microarray relative gene expression data: $\hat{\lambda_{i}}$	log_2_ ratio of two samples' microarray relative gene expression data: $\hat{\lambda_{i}}-\hat{\lambda_{j}}$, that is, (*a* − *b*)	log_2_ of qPCR relative gene expression data: ΔCt	log_2_ ratio of two samples' qPCR relative gene expression data: −ΔΔCt, that is, −(*a* − *b*)
GNG2	(a) L2A1 (b) L2B2	0.572 0.097	0.476	−0.35 0.14	0.49
	(a) L2B2 (b) L2A2	0.097 0.235	−0.139	0.14 0.80	0.66
	(a) L2A2 (b) L2D1	0.235 −0.389	0.625	0.80 1.75	0.95
	(a) L2D1 (b) L2C2	−0.389 −0.222	−0.167	1.75 0.50	−1.25
	(a) L2C2 (b) L2D2	−0.222 −0.258	0.035	0.50 1.21	0.71
	(a) L2D2 (b) L2D3	−0.258 0.131	−0.388	1.21 0.67	−0.54
	(a) L2D3 (b) L2A1	0.131 0.572	−0.442	0.67 −0.35	−1.02

RUNX3	(a) L2A1 (b) L2B2	0.835 −0.022	0.858	1.39 2.49	1.10
	(a) L2B2 (b) L2A2	−0.022 −0.042	0.020	2.49 2.81	0.32
	(a) L2A2 (b) L2D1	−0.042 −0.099	0.057	2.81 2.85	0.04
	(a) L2D1 (b) L2C2	−0.099 −0.258	0.159	2.85 2.37	−0.48
	(a) L2C2 (b) L2D2	−0.258 −0.190	−0.068	2.37 2.86	0.49
	(a) L2D2 (b) L2D3	−0.190 0.196	−0.385	2.86 2.37	−0.49
	(a) L2D3 (b) L2A1	0.196 0.835	−0.640	2.37 1.39	−0.98

CST7	(a) L2A1 (b) L2B2	1.301 0.224	1.076	−1.65 −0.90	0.75
	(a) L2B2 (b) L2A2	0.224 0.493	−0.268	−0.90 −0.72	0.18
	(a) L2A2 (b) L2D1	0.493 −0.454	0.946	−0.72 0.04	0.76
	(a) L2D1 (b) L2C2	−0.454 −0.414	−0.039	0.04 −0.01	−0.05
	(a) L2C2 (b) L2D2	−0.414 −0.325	−0.089	−0.01 0.23	0.24
	(a) L2D2 (b) L2D3	−0.325 0.300	−0.626	0.23 −0.62	−0.85
	(a) L2D3 (b) L2A1	0.300 1.301	−1.000	−0.62 −1.65	−1.03

MNDA	(a) L2A1 (b) L2B2	−1.136 0.313	−1.449	2.29 0.76	−1.53
	(a) L2B2 (b) L2A2	0.313 −0.229	0.542	0.76 1.88	1.12
	(a) L2A2 (b) L2D1	−0.229 −0.185	−0.044	1.88 1.31	−0.57
	(a) L2D1 (b) L2C2	−0.185 −0.015	−0.170	1.31 −0.08	−1.39
	(a) L2C2 (b) L2D2	−0.015 0.349	−0.365	−0.08 0.17	0.25
	(a) L2D2 (b) L2D3	0.349 0.357	−0.008	0.17 1.10	0.93
	(a) L2D3 (b) L2A1	0.357 −1.136	1.493	1.10 2.29	1.19

**Table 4:** The complete list of all enriched GO terms for 192 biomarker genes (sorted according to *P* value).

GO term	Description	# gene	*P* value
GO:0006952	Defense response	24	0.000000
GO:0006955	Immune response	24	0.000002
GO:0019882	Antigen processing and presentation	7	0.000073
GO:0009617	Response to bacterium	11	0.000088
GO:0006968	Cellular defense response	7	0.000149
GO:0001817	Regulation of cytokine production	11	0.000172
GO:0048534	Hemopoietic or lymphoid organ development	13	0.000562
GO:0030097	Hemopoiesis	12	0.000999
GO:0002520	Immune system development	13	0.001183
GO:0002696	Positive regulation of leukocyte activation	8	0.001499
GO:0001819	Positive regulation of cytokine production	7	0.001573
GO:0050867	Positive regulation of cell activation	8	0.002053
GO:0007610	Behavior	14	0.004162
GO:0002763	Positive regulation of myeloid leukocyte differentiation	4	0.004167
GO:0006412	Translation	12	0.004210
GO:0002684	Positive regulation of immune system process	10	0.006451
GO:0006414	Translational elongation	7	0.006467
GO:0002822	Regulation of adaptive immune response based on somatic recombination of immune receptors built from immunoglobulin superfamily domains	5	0.007075
GO:0048584	Positive regulation of response to stimulus	10	0.007697
GO:0001775	Cell activation	11	0.008090
GO:0051240	Positive regulation of multicellular organism process	10	0.008151
GO:0002819	Regulation of adaptive immune response	5	0.008161
GO:0030162	Regulation of proteolysis	5	0.008161
GO:0001818	Negative regulation of cytokine production	4	0.008578
GO:0002824	Positive regulation of adaptive immune response based on somatic recombination of immune receptors built from immunoglobulin superfamily domains	4	0.008578
GO:0002544	Chronic inflammatory response	3	0.009217
GO:0010829	Negative regulation of glucose transport	3	0.009217
GO:0050832	Defense response to fungus	3	0.009217
GO:0051043	Regulation of membrane protein ectodomain proteolysis	3	0.009217
GO:0002694	Regulation of leukocyte activation	8	0.009343
GO:0002706	Regulation of lymphocyte mediated immunity	5	0.009351
GO:0019725	Cellular homeostasis	13	0.009574
GO:0002821	Positive regulation of adaptive immune response	4	0.010479
GO:0042742	Defense response to bacterium	5	0.010649
GO:0042592	Homeostatic process	18	0.011731
GO:0006873	Cellular ion homeostasis	11	0.012021
GO:0050865	Regulation of cell activation	8	0.012239
GO:0009611	Response to wounding	15	0.012528
GO:0007267	Cell-cell signaling	15	0.013447
GO:0050778	Positive regulation of immune response	7	0.013518
GO:0031640	Killing of cells of another organism	3	0.013542
GO:0055082	Cellular chemical homeostasis	11	0.013819
GO:0002705	Positive regulation of leukocyte mediated immunity	4	0.014959
GO:0002708	Positive regulation of lymphocyte mediated immunity	4	0.014959
GO:0010827	Regulation of glucose transport	4	0.014959
GO:0034101	Erythrocyte homeostasis	5	0.015220
GO:0030098	Lymphocyte differentiation	6	0.016523
GO:0051251	Positive regulation of lymphocyte activation	6	0.016523
GO:0050801	Ion homeostasis	11	0.017255
GO:0045639	Positive regulation of myeloid cell differentiation	4	0.017545
GO:0002703	Regulation of leukocyte mediated immunity	5	0.018862
GO:0006935	Chemotaxis	7	0.020473
GO:0042330	Taxis	7	0.020473
GO:0045765	Regulation of angiogenesis	5	0.020868
GO:0006954	Inflammatory response	10	0.021243
GO:0046649	Lymphocyte activation	8	0.024760
GO:0045321	Leukocyte activation	9	0.026088
GO:0002761	Regulation of myeloid leukocyte differentiation	4	0.026706
GO:0050870	Positive regulation of T cell activation	5	0.030175
GO:0048872	Homeostasis of number of cells	6	0.030221
GO:0009620	Response to fungus	3	0.030551
GO:0032655	Regulation of interleukin-12 production	3	0.030551
GO:0031349	Positive regulation of defense response	5	0.032829
GO:0002699	Positive regulation of immune effectors process	4	0.033982
GO:0032103	Positive regulation of response to external stimulus	5	0.035615
GO:0048878	Chemical homeostasis	12	0.036704
GO:0002504	Antigen processing and presentation of peptide or polysaccharide antigen via MHC class II	3	0.037413
GO:0016525	Negative regulation of angiogenesis	3	0.037413
GO:0050850	Positive regulation of calcium-mediated signaling	3	0.037413
GO:0006928	Cell motion	13	0.037784
GO:0006334	Nucleosome assembly	4	0.037967
GO:0031497	Chromatin assembly	4	0.037967
GO:0048002	Antigen processing and presentation of peptide antigen	3	0.044801
GO:0010033	Response to organic substance	18	0.044896
GO:0030218	Erythrocyte differentiation	4	0.046616
GO:0065004	Protein-DNA complex assembly	4	0.046616
GO:0002521	Leukocyte differentiation	6	0.046793
GO:0014070	Response to organic cyclic substance	6	0.049526
GO:0002237	Response to molecule of bacterial origin	5	0.051544
GO:0007626	Locomotory behavior	8	0.052288
GO:0055080	Cation homeostasis	8	0.052288
GO:0050848	Regulation of calcium-mediated signaling	3	0.052676
GO:0051249	Regulation of lymphocyte activation	6	0.055270
GO:0034728	Nucleosome organization	4	0.056149
GO:0045619	Regulation of lymphocyte differentiation	4	0.056149
GO:0002697	Regulation of immune effectors process	5	0.058846
GO:0002712	Regulation of B cell mediated immunity	3	0.060999
GO:0002889	Regulation of immunoglobulin mediated immune response	3	0.060999
GO:0051235	Maintenance of location	4	0.061237
GO:0032914	Positive regulation of transforming growth factor-beta 1 production	2	0.061881
GO:0045651	Positive regulation of macrophage differentiation	2	0.061881
GO:0007611	Learning or memory	5	0.062693
GO:0008285	Negative regulation of cell proliferation	10	0.063328
GO:0051241	Negative regulation of multicellular organism process	6	0.064581
GO:0051789	Response to protein stimulus	5	0.066670
GO:0016192	Vesicle-mediated transport	13	0.067653
GO:0042060	Wound healing	7	0.068209
GO:0001906	Cell killing	3	0.069736
GO:0046635	Positive regulation of alpha-beta T cell activation	3	0.069736
GO:0051094	Positive regulation of developmental process	9	0.072195
GO:0050863	Regulation of T cell activation	5	0.075009
GO:0050900	Leukocyte migration	4	0.077725
GO:0009895	Negative regulation of catabolic process	3	0.078852
GO:0046634	Regulation of alpha-beta T cell activation	3	0.078852
GO:0030099	Myeloid cell differentiation	5	0.079368
GO:0055066	Di-, tri-valent inorganic cation homeostasis	7	0.080251
GO:0006323	DNA packaging	4	0.083611
GO:0030217	T cell differentiation	4	0.083611
GO:0043066	Negative regulation of apoptosis	10	0.085435
GO:0046324	Regulation of glucose import	3	0.088316
GO:0002863	Positive regulation of inflammatory response to antigenic stimulus	2	0.091380
GO:0032891	Negative regulation of organic acid transport	2	0.091380
GO:0032908	Regulation of transforming growth factor-beta 1 production	2	0.091380
GO:0051918	Negative regulation of fibrinolysis	2	0.091380
GO:0043069	Negative regulation of programmed cell death	10	0.092873
GO:0060548	Negative regulation of cell death	10	0.095435
GO:0045637	Regulation of myeloid cell differentiation	4	0.095934
GO:0030003	Cellular cation homeostasis	7	0.096877
GO:0045582	Positive regulation of T cell differentiation	3	0.098097

## Appendix

### A.

The microarray data of 4 biomarker genes from 7 samples were confirmed by quantitative PCR (qPCR). The correlation plots between microarray data and qPCR data are shown in Table 3. The correlation coefficients *r* for each gene are 0.73 (GNG2), 0.83 (RUNX3), 0.93 (CST7), and 0.76 (MNDA), shown in Figure 5.

### B.

The complete list of all enriched GO terms for 192 biomarker genes (sorted according to *P* value) is shown in Table 4.
